# 3D printing technology-assisted total hip arthroplasty for acute proximal femoral fracture and Hartofilakidis type II developmental dysplasia of the hip: a case report and literature review

**DOI:** 10.3389/fsurg.2026.1752462

**Published:** 2026-04-17

**Authors:** Hang Wen, Ming Liu, Bin Zhang, Yong Chen

**Affiliations:** Department of Orthopedic Surgery, Central Hospital Affiliated to Shenyang Medical College, Shenyang, Liaoning, China

**Keywords:** 3D printing, case report, developmental dysplasia of the hip, proximal femoral fracture, total hip arthroplasty

## Abstract

Developmental dysplasia of the hip (DDH) associated with an acute proximal femoral fracture on the same side is uncommon, and performing a one-stage total hip arthroplasty (THA) with concurrent fracture stabilization in such cases poses significant technical challenges. We present a case involving a 57-year-old female with a long history of right-sided DDH who was involved in a vehicular accident, leading to acute pain in her right hip, shortening of the limb, and restricted movement. Imaging studies indicated Hartofilakidis type II DDH along with a comminuted intertrochanteric fracture of the proximal femur and a pseudoacetabulum. Utilizing thin-slice computed tomography, we created a customized three-dimensional (3D) printed model of the pelvis and proximal femur, which allowed for detailed preoperative planning. This included evaluating the acetabular bone quality, identifying the true center of the acetabulum, selecting the appropriate cup size and orientation, and strategizing the femoral osteotomy and fixation with plates and cables. A one-stage cementless THA was executed through a posterolateral approach, featuring a small hemispherical cup securely placed in the true acetabulum and a size-16 biological femoral stem anchored distally across the fracture site, followed by the application of a lateral plate and titanium cable to stabilize the proximal femoral fracture. The patient began ambulation with the assistance of a walker on postoperative day 1. At 2 months after surgery, the pain score had decreased to 1/10 on the visual analog scale (VAS), and radiographic evaluation demonstrated ongoing fracture healing. By 3 months postoperatively, the patient was pain-free (VAS 0/10), had achieved a Harris Hip Score of 92, and showed restoration of lower-limb length. Imaging confirmed fracture union and stable prosthesis positioning, and the patient had returned to work independently. This case suggests that individualized 3D printing-assisted preoperative planning may improve the feasibility and early safety of one-stage cementless total hip arthroplasty combined with internal fixation for adult DDH with an ipsilateral proximal femoral fracture, and may provide a useful reference for preoperative decision-making in similarly complex cases.

## 1 Introduction

Developmental dysplasia of the hip (DDH) is characterized by abnormal development of the femoral head and acetabulum and may result in pain, functional impairment, and secondary osteoarthritis in adulthood, with some patients ultimately requiring total hip arthroplasty (THA) (1, 2). However, the complex anatomical abnormalities of the acetabulum and proximal femur in DDH make THA technically demanding and may increase the risk of prosthesis malposition, periprosthetic fracture, leg length discrepancy, and prolonged operative time (3, 4). For treating proximal femoral fractures, open reduction and internal fixation (ORIF) is typically used (5). Reports on the management of ipsilateral acute proximal femoral fracture in patients with DDH remain limited. Previous studies of 3D-assisted THA for DDH have mainly focused on elective procedures, particularly true acetabular identification, acetabular reconstruction, and acetabular cup positioning (6, 7), whereas its application in patients with concomitant acute proximal femoral fracture has rarely been described. In the present case, preoperative 3D planning was used not only for acetabular-side assessment, but also for evaluation of proximal femoral morphology, femoral stem selection, and fracture fixation planning. We therefore report this case to highlight the potential value of 3D-assisted preoperative planning in one-stage cementless THA combined with internal fixation for Hartofilakidis type II DDH with an ipsilateral acute proximal femoral fracture.

## 2 Case description

**Figure 1:**
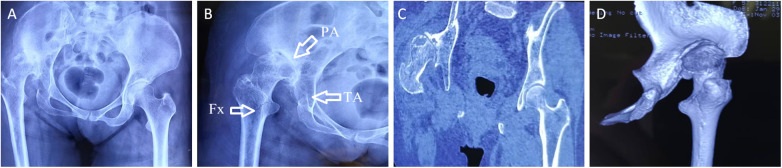
Preoperative x-ray and CT scans. **(A,B)** Preoperative radiographs showing superior displacement of the right femoral head associated with an ipsilateral acute proximal femoral fracture. In **(B)**, PA indicates the pseudoacetabulum, TA the true acetabulum, and Fx the fracture site. **(C,D)** CT images showing femoral head deformity, cystic changes, and pseudoacetabulum formation on the ilium.

**Figure 2:**
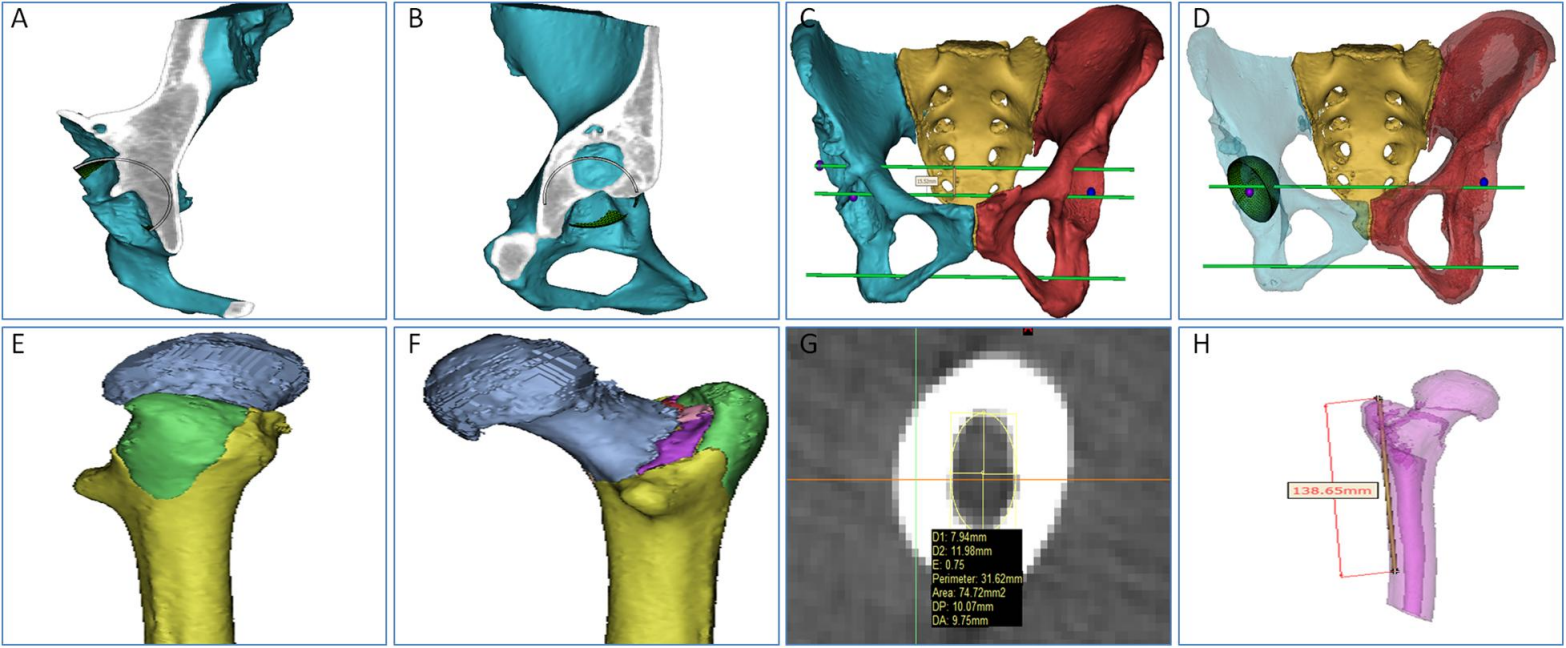
Preoperative 3D planning in MIMICS software. **(A,B)** Evaluation of acetabular bone quantity in sagittal and coronal planes. **(C,D)** Determination of the rotation center of the affected true acetabulum, implantation position, and size of the acetabular cup through the positioning of the healthy side acetabulum. **(E,F)** Simulation of reduction for the proximal femoral fracture. **(G,H)** Marking the site of femoral narrowing and the distance from the greater trochanter landmark.

**Figure 3:**
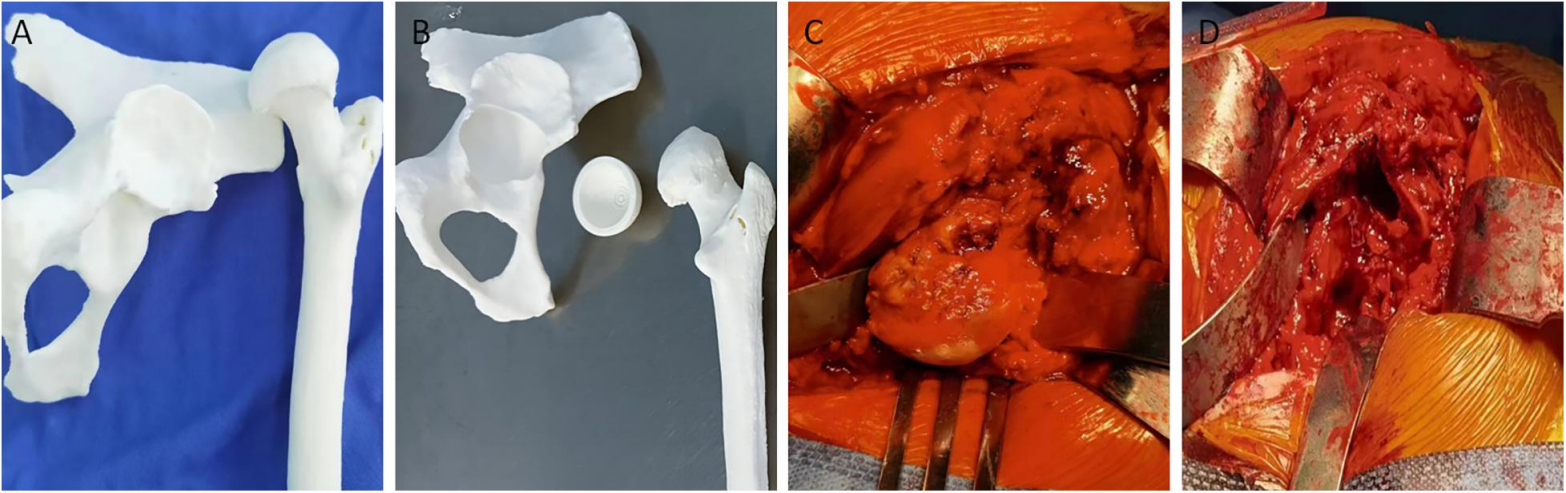
Patient's 3D model and intraoperative images. **(A)** 3D model of the right hip joint, with overlap of the true and pseudo acetabula. **(B)** 3D model after reaming on the true acetabulum. **(C,D)** THA removing the deformed femoral head and reaming the femoral medullary cavity.

A 57-year-old woman presented to our hospital 4 h after a traffic accident with pain in her right hip and restricted movement. Her medical history is generally unremarkable, with no major health issues. In infancy, she was diagnosed with developmental dysplasia of the hip (DDH) in her right hip, which later resulted in a limp, managed through conservative treatment. Upon examination, it was noted that her right leg was about 3 cm shorter than the left, and there was swelling in the right hip without skin compromise. Tenderness and positive axial percussion pain were observed in the affected area. Passive movement caused significant pain, hindering the assessment of the right hip's range of motion. Emergency x-rays indicated a continuous cortical disruption in the right proximal femur, accompanied by upward dislocation of the femoral head, localized collapse, cystic changes, and a narrowed joint space (Figures 1A, B). CT imaging confirmed a comminuted fracture in the right proximal femur, along with posterior and upward dislocation of the femoral head, deformity, local collapse, and the development of a pseudoacetabulum on the ilium, showing partial overlap between the true and false acetabula (Figures 1C, D). To improve the preoperative evaluation of the acetabulum and proximal femur, we imported the preoperative CT data in DICOM format into MIMICS software. We created a three-dimensional model of the pelvic bone to analyze the position, size, and shape of both acetabula, evaluate the extent and location of bone defects, and plan the acetabular reaming's position, angle, and depth. Furthermore, we determined the diameter, anteversion angle, and abduction angle for the acetabular cup to be utilized during the procedure (Figures 2A–D). All fragments of the proximal femur were labeled, and a simulated reduction of these fragments was conducted (Figures 2E, F). The proximal femur was assessed for deformities, and the dimensions of the narrowed medullary cavity and estimated size of the femoral shaft were recorded (Figures 2G, H). The three-dimensional modeling data were exported in STL format and imported into FashPrint 5 software (ShanZuan Technology, China) to create customized 1:1 models of the pelvis, acetabulum post-reaming, and proximal femur (Figures 3A, B).

Following the administration of general anesthesia, the patient was positioned on her left side, and a posterolateral approach was utilized for the hip joint surgery. An incision was made through the skin, subcutaneous tissue, and fascia. The gluteus maximus was separated bluntly, and the external rotator muscles were severed at the greater trochanter. The posterior joint capsule was cut, exposing the right femoral head, which was dislocated both posteriorly and superiorly, appearing collapsed and flattened. A pseudoacetabulum had developed on the ilium, while the proximal femur exhibited a comminuted fracture with several small fragments. The bone was sectioned about 1.5 cm above the lesser trochanter, and the femoral head was excised. The true acetabulum was located using the acetabular fossa, remnants of the round ligament, and a 3D printed model as references. Beneath the false acetabulum, a triangular true acetabulum was identified, filled with adipose tissue and showing signs of osteoporosis. Utilizing the 3D printed pelvic model, the hip joint's rotation center was pinpointed, and the bony limits of the true acetabulum were revealed. The position, depth, anteversion angle, and abduction angle for acetabular reaming were established. The true acetabulum was reamed until 80% of the cancellous bone was visible with point bleeding, ensuring an anteversion angle of 20 degrees and an abduction angle of 45 degrees. A size 44 biological acetabular cup was press-fitted, and three cancellous bone screws were placed in the upper outer quadrant. A suitably sized polyethylene liner was inserted, and cancellous bone from the femoral head was positioned above the acetabular prosthesis. The proximal femur was exposed, and the medullary canal was accessed. Following the preoperative plan with the 3D model, a properly sized femoral stem trial was placed after achieving satisfactory reduction. A size-16, distally fixed biological femoral stem was then implanted, along with a standard ceramic head prosthesis. Post-reduction, the hip joint demonstrated good mobility without dislocation. The proximal femoral fracture was reduced, and a plate was affixed to the lateral aspect of the femur using screws and titanium cables for stabilization. After standard hemostasis and irrigation, the incision was closed in layers.

**Figure 4:**
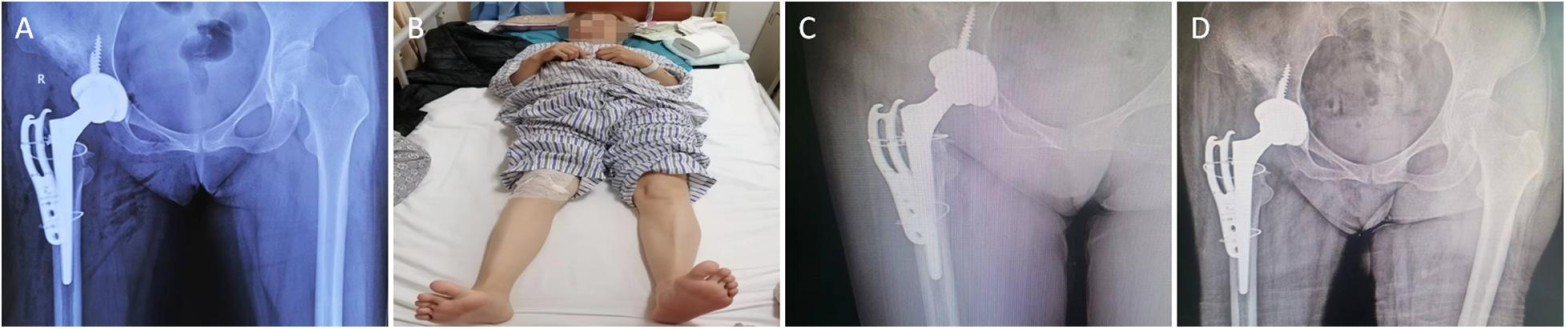
Postoperative x-ray images and external appearance. **(A)** x-ray images of the patient's right hip THA 1 day postoperatively, showing the femoral fracture line. **(B)** Both lower limbs are of equal length on the first day after surgery. **(C)** x-ray examination of the patient's THA 2 months postoperatively, with the proximal femoral fracture line becoming less distinct. **(D)** The x-ray shows that the proximal femoral fracture has healed, and the prostheses are in good positions, 3 months postoperatively.

Following the surgery, the patient underwent a regimen focused on alleviating pain, facilitating the healing of the fracture, preventing infections, and reducing the risk of deep vein thrombosis in the legs. On the day after the operation, x-ray results showed appropriate abduction and anteversion angles of the acetabular cup, with the centers of rotation for the femoral heads on both sides being level. The femoral stem was correctly aligned within the medullary canal, the proximal fragments of the femur were properly reduced, and the installation of the plate, screws, and titanium cables was deemed satisfactory (Figure 4A). Assessments post-surgery indicated that both legs were of equal length, and there were no indications of numbness (Figure 4B). The patient was able to walk with a walker on the first day after surgery. During the follow-up at two months, the patient reported only slight discomfort, with a visual analog scale (VAS, 0–10; where 0 means no pain and 10 represents the worst pain) score of 1/10. x-rays showed that the hip prosthesis and plate were well-placed, and the fracture line was less distinct (Figure 4C). At the three-month follow-up, x-rays confirmed the proper positioning of the prosthesis and indicated that the fracture had healed (Figure 4D). Clinically, the patient experienced no pain (VAS 0/10), and her Harris hip score was 92, reflecting excellent hip functionality. She was able to walk unaided and had returned to her job. The patient expressed great satisfaction with the results, saying: “After years of limping and after this accident, I was worried that I would be disabled. Now I am pain-free, my legs are equal in length, and I returned to work within three months. I feel as if I have a new hip.” A detailed timeline of the treatment process is provided in Supplementary Table S1.

## 3 Discussion

Developmental dysplasia of the hip (DDH) may lead to secondary osteoarthritis and femoral head necrosis in adults (8). It typically presents with hip pain and limping, often impairing daily function. For patients with advanced DDH-related arthritis, total hip arthroplasty (THA) is an effective treatment to restore hip function (7, 9). Hartofilakidis classified DDH into three types according to the radiographic relationship between the true acetabulum and the femoral head (10). In type II DDH, the acetabulum is often small and shallow, and a pseudoacetabulum usually forms above the true acetabulum after dislocation. Bone deficiency is common at the junction of the lower pseudoacetabulum and the upper true acetabulum, and superolateral acetabular bone stock decreases as dislocation progresses (11). Therefore, THA in Hartofilakidis type II DDH is more challenging than in routine arthritic hips, with increased risks of insufficient cup coverage, postoperative loosening or dislocation, and later revision (12). To restore hip biomechanics and acetabular stability, several techniques have been proposed, including acetabular roof reconstruction, medialization, use of a small cup, and elevation of the hip center.

Techniques for reconstructing the acetabular roof mainly consist of bone grafting (13) and metal block methods (14). These methods can improve acetabular prosthesis coverage, enhance initial stability, and help restore the anatomical hip center. However, bone grafting may lead to graft resorption and collapse, thereby increasing the risk of acetabular loosening (15). In DDH with non-contained bone defects, metal augments may have limited adaptability to the acetabular bone bed and are more commonly used in revision surgery for acetabular defects (16). The medial wall protrusion technique involves actions such as grinding, cutting, and fracturing the medial wall to enhance cup coverage, aiming for long-term stability (17), but excessive medialization may reduce acetabular bone stock, alter stress distribution, and compromise stability (18). The small cup technique uses a smaller component to match the shallow true acetabulum and improve coverage, but its limited diameter and load-bearing area may increase stress concentration and the risk of dislocation (19, 20). The rotation center elevation technique involves positioning the acetabular cup higher to enhance host bone coverage, simplifying the procedure by reconstructing the acetabulum on the pseudoacetabulum (21). However, this upward adjustment can lead to issues such as compromised abductor function, discrepancies in leg length, abnormal gait, and a heightened risk of prosthetic loosening (22). In conclusion, there are various biomechanical pros and cons associated with acetabular reconstruction techniques for patients with Hartofilakidis type II DDH, and a standardized criterion is currently lacking.

This case involved Hartofilakidis type II developmental dysplasia of the hip (DDH) complicated by an ipsilateral acute comminuted proximal femoral fracture. The main challenge was to achieve true acetabular reconstruction and stable femoral fracture fixation in a single-stage procedure. Because DDH is associated with complex deformities of the acetabulum, femur, and surrounding soft tissues, and the acute fracture further complicates femoral-sided management, conventional imaging alone may not fully delineate the local three-dimensional anatomy. This increases the technical difficulty of acetabular reconstruction, soft-tissue balancing, and femoral preparation, and may raise the risks of prosthesis malposition, periprosthetic fracture, leg length discrepancy, and prolonged operative time. The value of this case lies in the use of 3D-assisted planning in a complex setting involving both hip deformity and acute fracture. Previous reports have mainly focused on elective total hip arthroplasty (THA) for DDH, particularly true acetabular identification, acetabular reconstruction, and prosthesis positioning, whereas the present case also required simultaneous management of an acute proximal femoral fracture. Preoperative 3D modeling helped assess the relationship between the true and false acetabula, estimate acetabular bone stock, simulate cup placement, and evaluate proximal femoral deformity, canal narrowing, and fracture morphology, thereby guiding femoral stem selection and fracture fixation strategy. This made preoperative planning more individualized and the intraoperative procedure more predictable.

In recent years, the development of 3D printing technology has led to its increasing use in orthopedic preoperative planning because of its intuitive three-dimensional visualization (23). Previous studies have suggested that 3D-assisted planning for total hip arthroplasty (THA) may shorten operative time, reduce blood loss, improve surgical accuracy, and enhance patient understanding and compliance (24). In the present case, CT data were imported into Mimics 20.0 software to reconstruct three-dimensional models of the pelvis and proximal femur. Preoperative evaluation of the true acetabulum, surrounding bone stock, femoral canal morphology, and anteversion helped us select appropriate acetabular and femoral components and may have reduced the risk of periprosthetic fracture caused by forceful implantation. Femoral stem selection in THA for developmental dysplasia of the hip (DDH) remains controversial and is largely influenced by combined anteversion and the coronal and axial morphology of the femur (25). In this context, CT-based three-dimensional preoperative planning may be valuable for stem evaluation and selection. A recent biomechanical study by Shah et al. indicated that the mechanical strength and load transfer of the proximal femur are closely associated with the medial cortex, calcar femorale, and the medial and lateral trabecular systems, whereas the intertrochanteric region and the anterolateral and posterolateral aspects of the femoral neck are relatively vulnerable areas (26). Based on this biomechanical consideration and the compromised proximal bony support in the present case, we selected a distally fixed biological femoral stem to reduce reliance on proximal cancellous bone for initial fixation and obtain more reliable axial fixation distal to the main fracture zone. Preoperative three-dimensional planning also helped assess femoral canal morphology and anticipate stem trajectory and anteversion, thereby facilitating intraoperative decision-making. Notably, the acetabular and femoral implant sizes used intraoperatively were consistent with the preoperative plan, and no intraoperative fracture occurred on either the femoral or acetabular side, which may support the potential utility of 3D-assisted preoperative planning in similarly complex cases. Previous studies have also shown that preoperatively selected acetabular cup sizes on 3D models in DDH may match the actual implants used intraoperatively in up to 71.4% of cases (27). Precise preoperative planning enabled appropriate prosthesis selection and reduced the need for repeated intraoperative adjustment. This may help shorten operative time, reduce blood loss, and lower the risk of complications such as infection and periprosthetic fracture.

Beyond implant selection, 3D printing-assisted preoperative planning may help address several common pitfalls of THA in patients with DDH. First, it may improve identification of the true acetabulum relative to the false acetabulum, thereby reducing the risk of cup malposition. Second, it allows preoperative estimation of acetabular bone stock and superolateral coverage, which may assist in selecting an appropriately sized small acetabular component and avoiding insufficient host bone coverage. Third, simulation of femoral preparation and implant positioning may provide a useful reference for limb-length restoration and potentially reduce the risk of postoperative leg length discrepancy. In the present case, which was further complicated by an acute proximal femoral fracture, the three-dimensional model also facilitated a more intuitive understanding of the spatial relationship between the dysplastic femur and the fracture fragments. This may help reduce the risk of inappropriate stem selection, intraoperative dislocation, and periprosthetic femoral fracture related to abnormal femoral morphology, increased femoral neck anteversion, and fracture-related interference. Such advantages may be particularly relevant in cases involving both deformity and trauma, where conventional two-dimensional imaging may underestimate the complexity of the local three-dimensional anatomy.

We explained to the patient and her family that two surgical options were available. The first was a single-stage procedure combining open reduction and internal fixation (ORIF) of the proximal femoral fracture with total hip arthroplasty (THA) for developmental dysplasia of the hip (DDH). Although this approach could address both conditions simultaneously, it involved a longer operative time and increased risks of infection and non-union, potentially resulting in surgical failure. The second option was staged treatment, with initial ORIF followed by THA after fracture healing. Although considered safer, this strategy required two operations, with more scarring and higher cost. After careful discussion, the patient and her family chose the single-stage procedure to avoid the additional trauma and financial burden of two separate surgeries. In accordance with their preference and the principles of enhanced recovery after surgery (ERAS) (28), we used 3D printing for preoperative planning and simulated fracture reduction. After implantation of a distally fixed biological femoral stem, the fracture was reduced and stabilized with a lateral plate, screws, and titanium cables. The distally fixed stem allowed weight-bearing and ambulation on postoperative day 1, facilitating early rehabilitation and reducing complications associated with prolonged bed rest, such as pressure ulcers, pulmonary infection, urinary tract infection, deep vein thrombosis, and muscle atrophy.

Furthermore, patient-specific 3D-printed pelvic and proximal femoral models allowed comprehensive assessment of acetabular wall bone quality, the locations of the true and false acetabula, femoral morphology, and femoral canal narrowing. These models also enabled simulation of key procedures, including acetabular reaming, femoral canal preparation, reduction, and prosthesis placement. Such practice may aid the training of less experienced surgeons, improve communication with patients and their families, and promote patient participation in surgical decision-making. By reducing patient anxiety and stress, this approach is consistent with ERAS principles and may support postoperative recovery, improve surgical outcomes, and reduce complications.

The application of 3D printing in total hip arthroplasty (THA) and femoral fracture fixation for developmental dysplasia of the hip (DDH) remains challenging because of the additional cost and time required for model design and printing. The accuracy of bone segmentation also depends on the quality of CT acquisition and image-processing expertise, which may lead to discrepancies between the printed model and actual anatomy. In addition, patient-specific guides and navigation technologies were not used in this study, representing a further limitation. Future integration of these techniques may improve intraoperative reproducibility and accuracy in patients with complex anatomical abnormalities. Nevertheless, no significant perioperative or postoperative complications were observed in this case. The patient experienced marked pain relief and functional improvement, returned to work, and achieved fracture healing by 3 months, although the follow-up period remains short and long-term outcomes are still unknown. In conclusion, 3D printing-assisted THA combined with internal fixation may facilitate acetabular and femoral evaluation, support surgical planning, promote fracture healing and functional recovery, and demonstrate acceptable early safety. This case report serves to offer orthopedic surgeons a viable treatment option for acute proximal femoral fractures associated with Hartofilakidis type II DDH.

## Data Availability

The original contributions presented in the study are included in the article/Supplementary Material, further inquiries can be directed to the corresponding author.
