# Supplementary material for: 3D printing technology-assisted total hip arthroplasty for acute proximal femoral fracture and Hartofilakidis type II developmental dysplasia of the hip: a case report and literature review
Source: Front Surg. 2026 Apr 17;13:1752462. doi: 10.3389/fsurg.2026.1752462 (PMC13132816; doi:10.3389/fsurg.2026.1752462)
Supplement: Supplementary file 1 [file Table1.docx]

Supplementary Table S1. Timeline of the clinical course.

| **Time point** | **Event or intervention** | **Main clinical findings** |
| --- | --- | --- |
| 4 h after injury (on admission) | Presentation to the emergency department after a vehicle collision | Acute right hip pain, limb shortening and restricted movement; history of right-sided DDH since infancy managed conservatively. |
| On admission | Initial clinical and radiological assessment | Right leg shorter than the left, swelling and tenderness around the right hip, severe pain with passive motion. X-rays and CT revealed a comminuted proximal femoral (intertrochanteric) fracture with posterosuperior dislocation of the femoral head, Hartofilakidis type II DDH and pseudoacetabulum formation. |
| Preoperative period (index hospitalization) | 3D image processing and printing-based preoperative planning | CT data were imported into dedicated software to construct 3D models of the pelvis and proximal femur. The true acetabulum, bone defects, planned cup position, and femoral canal morphology and anteversion were assessed, simulated fragment reduction and prosthesis templating were performed, and 1:1 pelvic and proximal femoral models were 3D-printed. |
| Index surgery (same hospitalization) | One-stage cementless THA combined with internal fixation of the proximal femoral fracture | Via a posterolateral approach, the true acetabulum was prepared and a press-fit cup with supplemental screw fixation and cancellous bone graft from the femoral head was implanted according to the preoperative 3D plan. A distally fixed biological femoral stem and ceramic head were inserted. The comminuted proximal femoral fracture was reduced and stabilized with a lateral plate and titanium cables. |
| Postoperative day 1 | Early postoperative assessment and mobilization | Radiographs showed proper cup orientation, correct stem alignment, satisfactory reduction of proximal femoral fragments and stable plate/cable fixation. Leg lengths were equal and no numbness was noted. The patient initiated walking with a walker on postoperative day 1. |
| Postoperative 2-month follow-up | Outpatient review with radiographs and clinical assessment | The patient reported only mild discomfort in the right hip (low VAS score). Radiographs demonstrated good positioning of the hip prosthesis and plate with a less distinct fracture line, indicating progression of fracture healing. |
| Postoperative 3-month follow-up | Outpatient review with radiographs and functional evaluation | Radiographs confirmed fracture union and stable prosthetic components. The patient was pain-free, had an excellent Harris hip score, walked without support, returned to work, and had equal leg length and satisfactory hip function. |
